# Supplementary material for: A network dynamical simulation model for the study of antibiotic resistance in microbial communities
Source: Gut Microbes. 2026 Sep 19;18(1):2734703. doi: 10.1080/19490976.2026.2734703 (PMC13596919; doi:10.1080/19490976.2026.2734703)
Supplement: Supplementary file 1 [file KGMI_A_2734703_SM9053.zip › supplementary_file_1/Figures/er_delta_gc_size_n1000.pdf]

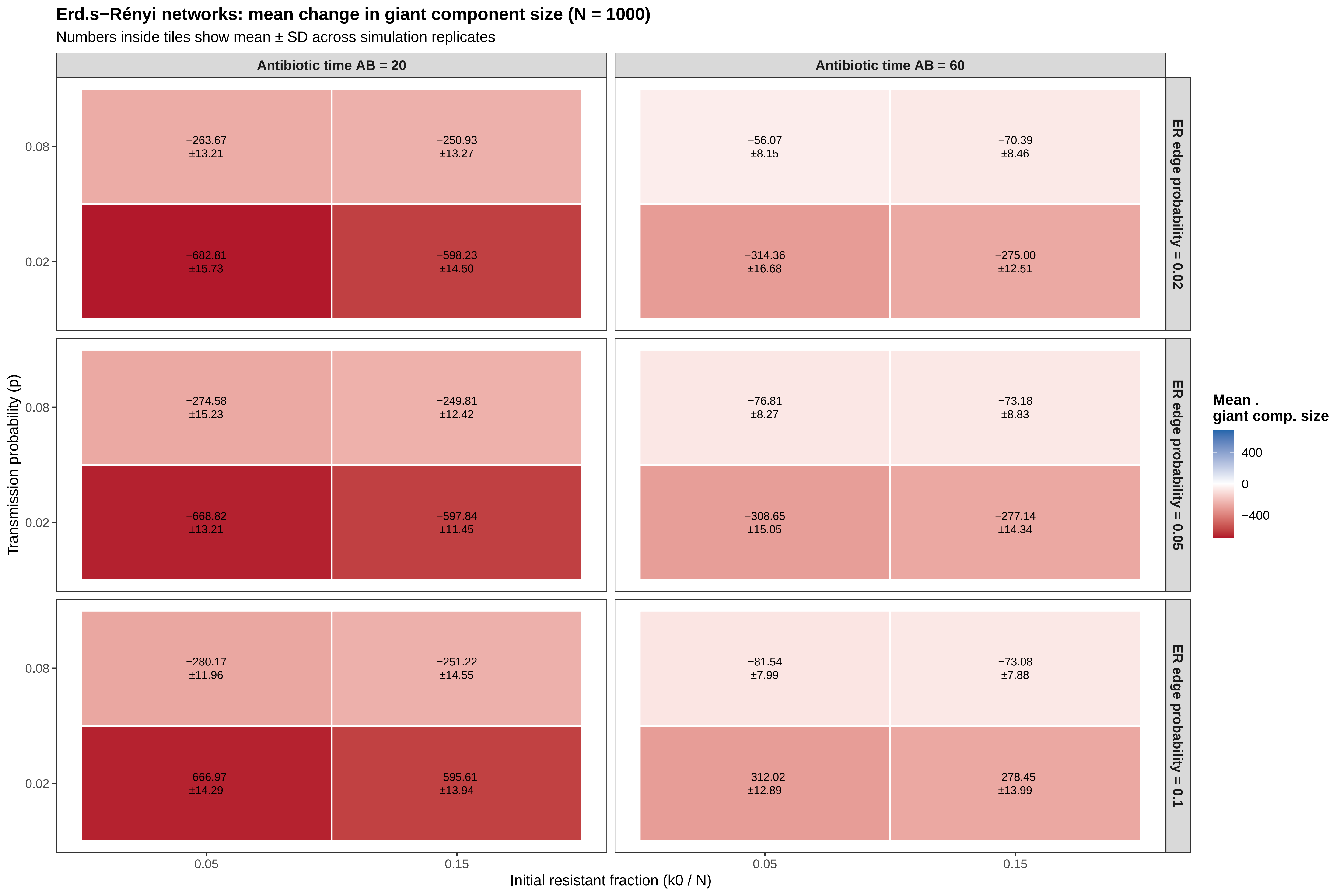

Erd.s−Rényi networks: mean change in giant component size (N = 1000)
Numbers inside tiles show mean ± SD across simulation replicates
Antibiotic time AB = 20
Antibiotic time AB = 60
ER edge probability = 0.02
ER edge probability = 0.05
ER edge probability = 0.1
−263.67 ±13.21
−250.93 ±13.27
−682.81 ±15.73
−598.23 ±14.50
−56.07 ±8.15
−70.39 ±8.46
−314.36 ±16.68
−275.00 ±12.51
−274.58 ±15.23
−249.81 ±12.42
−668.82 ±13.21
−597.84 ±11.45
−76.81 ±8.27
−73.18 ±8.83
−308.65 ±15.05
−277.14 ±14.34
−280.17 ±11.96
−251.22 ±14.55
−666.97 ±14.29
−595.61 ±13.94
−81.54 ±7.99
−73.08 ±7.88
−312.02 ±12.89
−278.45 ±13.99
0.08
0.02
0.05
0.15
Transmission probability (p)
Initial resistant fraction (k0 / N)
Mean .
giant comp. size
400
0
−400
